# Fetuin-A mediates the difference in adipose tissue insulin resistance between young adult pakistani and norwegian patients with type 2 diabetes

**DOI:** 10.1186/s12902-022-01127-y

**Published:** 2022-08-17

**Authors:** Sindre Lee-Ødegård, Thor Ueland, Per M. Thorsby, Pål Aukrust, Annika E. Michelsen, Bente Halvorsen, Christian A. Drevon, Kåre I. Birkeland

**Affiliations:** 1 Institute of Clinical Medicine, University of Oslo, Oslo, Norway; 2 Research Institute of Internal Medicine, Oslo University Hospital, Oslo, Norway; 3 Hormone Laboratory, Dep of Medical Biochemistry and Biochemical endocrinology and metabolism research group, Oslo University Hospital, Aker, Oslo Norway; 4 Department of Nutrition, Institute of Basic Medical Sciences, Faculty of Medicine, University of Oslo, Oslo, Norway; 5 Department of Endocrinology, Morbid Obesity and Preventive Medicine, Oslo University Hospital, Oslo, Norway

**Keywords:** Hepatokine, Adipose tissue, fetuin-A, Free fatty acids, Ethnicity, Insulin sensitivity, Clamp

## Abstract

**Background:**

South-Asian immigrants to Western countries have a high prevalence of type 2 diabetes mellitus (T2DM) and increased adipose tissue insulin resistance (AT-IR), as compared to their Western counterparts. Fetuin-A is a hepatokine known to influence AT-IR.

**Aim:**

Can plasma fetuin-A concentrations explain an ethnic difference in adipose tissue insulin resistance?

**Methods:**

We performed a two-step euglycemic-hyperinsulinaemic clamp and measured plasma concentrations of fetuin-A and non-esterified fatty acids (NEFA), in 18 Pakistani and 21 Norwegians with T2DM (age 29–45y) in Norway. AT-IR was calculated as NEFA-suppression during the clamp. The adipokines/cytokines leptin, adiponectin, visfatin, PTX3, IL-1β, INF-γ, and IL-4 were measured in fasting plasma. Liver fat was estimated by CT-scans.

**Results:**

Despite a lower BMI, Pakistani patients displayed higher AT-IR than Norwegians. NEFA-suppression during clamp was lower in Pakistani than Norwegians (mean=-20.6%, 95%CI=[-40.8, -0.01] and *p* = 0.046). Plasma fetuin-A concentration was higher in Pakistani than Norwegians (43.4 ng/mL[12.7,74.0], *p* = 0.007) and correlated negatively to %NEFA-suppression during clamp (rho=-0.39, *p* = 0.039). Plasma fetuin-A concentration explained 22% of the ethnic difference in NEFA-suppression during the clamp. Pakistani patients exhibited higher plasma leptin and lower PTX3 levels than Norwegian, and plasma visfatin correlated positively to plasma fetuin-A levels in the Pakistani patients. We observed no correlation between plasma fetuin-A and liver fat, but fetuin-A correlated negatively with plasma IL-1β, INF-γ, and IL-4 concentrations. Plasma IL-4 concentration was lower in Pakistani than in Norwegian patients.

**Conclusion:**

Fetuin-A may contribute to explain the discrepancy in T2DM prevalence between Pakistani and Norwegians patients by influencing AT-IR.

## Introduction

South Asian immigrants living in Western countries have a high prevalence of type 2 diabetes mellitus (T2DM) [1–6]. Norway has a growing population of Pakistani immigrants, especially in the capital Oslo, with a high prevalence of T2DM manifesting at a younger age than in ethnic Norwegians [7]. The pathophysiology of T2DM in this population may differ from the common form of T2DM in the Western population, and a deeper understanding may help to develop more efficient prevention and treatment strategies, and also give new insight into the general pathogenesis of T2DM [8]. For example, our previous findings indicated a more pronounced influence of insulin resistance on lipid metabolism in Pakistani compared to Norwegian patients with T2DM [9].

Expanded and inflamed adipose tissue is a major contributor to insulin resistance in other tissues, such as the liver [10, 11], by increased lipolysis and altered adipokine secretion [12]. However, a reverse relationship may also exist, where the liver induces adipose tissue resistance through altered secretion of hepatokines [13, 14]. This cross-relationship is known as the “adipose tissue-liver axis” of insulin resistance [13].

Recently, several studies have focused on the hepatokine fetuin-A and its potential role in regulating adipose tissue insulin resistance [15–20]. In vitro studies have shown that fetuin-A may serve as an adaptor protein for saturated fatty acid (SFA) and explain SFA-induced activation of Toll-like receptor 4 (TLR4) signaling and inhibition of the insulin receptor tyrosine kinase in adipocytes [19–21]. Furthermore, rodents lacking fetuin-A may be protected from diet-induced insulin resistance [22]. In humans, high levels fetuin-A and nonesterified fatty acids (NEFA) may together predict insulin resistance in adipose tissue [15, 20]. Moreover, fetuin-A has been shown to impair insulin receptor tyrosine kinase activity [21]. Notably, fetuin-A mediated adipose tissue insulin resistance may promote inflammatory cytokine production [19]. Obese human participants with T2DM have increased levels of fetuin-A [19] that correlate with increased levels of several pro-inflammatory cytokines [23]. Furthermore, fetuin-A may inhibit adiponectin production in adipocytes, contributing to insulin resistance [24]. However, it remains unknown if ethnic differences in adipose tissue insulin resistance [9] can be explained by altered secretion of fetuin-A.

We hypothesized that (i) fetuin-A would be associated with adipose tissue insulin resistance in T2DM, and (ii) could mediate ethnic differences in T2DM prevalence between young Pakistani and Norwegians. We have measured plasma concentration of fetuin-A and adipose tissue insulin resistance, calculated as NEFA-suppression during a two-step euglycemic hyperinsulinemic clamp, in young Pakistani and Norwegian men and women with T2DM from the DIPI study [25].

## Materials and methods

DIPI is a cross-sectional study comparing young first generation Pakistani immigrants with T2DM living in Oslo, Norway to ethnic Norwegian patients aged ≤ 45 years [9, 26]. Exclusion criteria were: ethnicities other than Norwegian or Pakistani, positive antibodies to glutamic acid decarboxylase (GAD) or islet antigen 2 (IA2) auto-antibodies, age > 45 years, persons unwilling or unable to give informed consent.

### Participants

Details regarding the study participants had been described previously [9, 26]. Briefly, T2DM patients of either Pakistani (*n* = 18) or Norwegian (*n* = 21) origin (51% women) between age 29 to 45 years were recruited from the diabetes outpatient clinics at Lovisenberg Deaconess Hospital and Aker University Hospital in Oslo. We searched patient registries and identified 195 patients that were randomly invited to participate in the study. All participants were treated according to current national and international guidelines for T2DM with education about healthy lifestyle and pharmacological antidiabetic treatment as appropriate. None were treated with GLP-1 receptor agonists or SGLT2-inhibitors. Participant characteristics were similar to a subgroup of 80 with available data out of the 155 non-included patients in terms of HbA1cand anthropometrics.

### Anthropometrical measurements

Height and weight were measured with light clothing without shoes. Waist and hip circumferences were assessed with a tape at the midpoint between the lowest rib margin and the iliac crest, and at the level of the major trochanter, respectively. A Tanita Body Composition Analyzer BC-418 (Tokyo, Japan) was used to estimate percentage total body fat, body fat mass in kilograms and fat free mass in kilograms from a subset of Norwegians (*n* = 17) and Pakistani (*n* = 14) patients. A CT Somatom (Erlangen, Germany) scan was performed on patients in a supine position with their arms above the head. Liver fat content was estimated from attenuation in Hounsfield Units (HU). All subjects were fasting and voided urine before measurement.

### Insulin resistance

All patients stopped taking oral antidiabetic drugs for two days, and insulin for at least 12 h before the examination [9, 26]. They also refrained from strenuous physical exercise and alcohol intake during these two days, and they fasted from midnight before the examination. We performed a euglycemic hyperinsulinemic clamp [27] with two steps; first administering a primed, continuous insulin dose of 40 mU/m^2^/min for a minimum of 100 min, until at least 30 min of stable euglycemia was obtained, and then immediately a 400 mU/m^2^/min insulin infusion, also for a minimum of 100 min, with at least 30 min of stable euglycemia at the end. Body surface area was calculated using the Mosteller equation [28]. Insulin sensitivity was measured as the glucose infusion rate during low and high insulin infusion rates in µmol/m^2^/min and denoted GIR_40_ and GIR_400_, respectively.

EDTA plasma was collected for non-esterified fatty acid (NEFA) measurements before and 30 min into the two steps euglycemic clamp. EDTA plasma was immediately frozen at -70° C after collection. Adipose tissue insulin resistance was defined as percent NEFA-suppression from before to 30 min into the clamp [9].

### Biomarker analysis

Fasting plasma glucose was measured by the glucose oxidase method on a Glucose Analyzer II (Beckman Instruments, CA 92,821, USA). Serum insulin was analyzed using the radioimmunoassay (RIA) kit, formerly from Linco Research Inc. (St. Charles, MO, USA), presently available from Millipore Corp. (Billerica, MA, USA). HbA_1c_ was measured by high-performance liquid chromatography on a Variant analyzer (Bio-Rad, Richmond, CA, USA). Fasting serum total cholesterol, HDL cholesterol, and triglycerides were measured using a routine enzymatic method (Roche Diagnostics, Mannheim, Germany). Serum LDL cholesterol was calculated using the Friedewald equation [29]. NEFA were analyzed using a NEFA C enzymatic color test kit, (Wako Chemicals GmbH, Neuss, Germany), modified to run on a Technicon RA1000 (Technicon Instruments Corp., Tarrytown, NY). Plasma levels of adiponectin and leptin were analyzed using RIA kits from Millipore Corp. (Billerica, MA, USA). Measurements of plasma levels of fetuin-A were performed using Human fetuin-A ELISA (BioVendor Laboratory Medicine, Inc. (Modrice, Czech Republic). Pentraxin 3 (PTX3) was measured using ELISA from R&D systems (Minneapolis, MN, USA). IL-1β, INF-γ and IL-4 were assessed using BioPlex Human Cytokine panel, Bio-Rad Laboratories Inc (Hercules, CA, USA). The intra-assay variability for fetuin-A was on average 3.75% between duplicates. Only one assay was used so there is no inter-assay variability issue.

### Power calculations

Post-hoc power analyses indicated that we achieved 91.5% power to detect ethnical difference in plasma fetuin-A levels, and 71.1% power to detect ethnical difference in NEFA suppression during the clamp.

### Statistical analyses

Group comparisons are presented as box-and-whisker plots and statistical significance was calculated using Wilcoxon signed-rank tests. Bivariate and partial (correction for BMI) correlations were tested using Spearman’s ranked tests and presented as scatter plots. Mediation analyses were performed using Baron and Kenny’s mediation steps [30]. Briefly, the null model was a linear model for adipose insulin resistance (the outcome) as a function of ethnicity. Next, a model was constructed substituting the outcome with the mediator of interest (plasma fetuin-A concentration). Finally, a full model was constructed similar to the null model, but also included the mediator of interest as a covariate. The potential interpretations of the full model are as follows [30]: (1) if the effect of ethnicity on adipose tissue insulin resistance is reduced and becomes insignificant, the mediator (fetuin-A) may fully explain ethnic differences in adipose tissue insulin resistance; (2) if the effect of ethnicity on adipose tissue insulin resistance is reduced, but remains significant, the mediator (fetuin-A) may only partially explain ethnic differences in adipose tissue insulin resistance, and; (3) if the effect of ethnicity on adipose tissue insulin resistance remains similar despite the presence of the potential mediator, then fetuin-A does not explain ethnic differences in adipose tissue insulin resistance. We considered a *P* ≤ 0.05 as statistically significant. R 3.1.1 was used for all calculations.

## Results

### Group differences at baseline

The groups were similar in terms of age, sex, and fasting plasma glucose, as described previously [9]. Norwegian patients exhibited higher BMI, body weight, total body fat mass, and lean body mass, and had a shorter duration of diabetes as compared to Pakistani (Table 1).

**Table 1:** Clinical characteristics of patients of Norwegian and Pakistani ethnicity

	Norwegian (*n* = 21)	Pakistani (*n* = 18)
Age (years)	42 (6)	41 (8)
BMI (kg/m^2^)	37.2 (6.0)	30.9 (9.4)**
Fasting C-peptide (pmol/L)	1162 ± 458	977 ± 373
Fasting insulin (pmol/L)	166 (160)	209 (193)
Fasting plasma glucose (mmol/L)	10.7 ± 3.2	10.6 ± 3.3
HbA1c (% mmol/mol)	7.3/56 (1.4/13)	8.7/72 (2.9/31)*
HDL cholesterol (mmol/L)	1.03 ± 0.21	1.08 ± 0.24
LDL cholesterol (mmol/L)	2.7 ± 0.8	2.6 ± 0.6
Lean body mass (kg)	67.1 ± 12.6	54.2 ± 12.2**
Men (%)	10 (48%)	9 (50%)
Total body fat (%)	36.9 ± 9.6	34.2 ± 7.7
Total body fat mass (kg)	39.5 ± 12.0	28.0 ± 8.2**
Thigh area (AU)	337 ± 57.7	294 ± 65.8*
Thigh SAT	165 ± 64.3	137 ± 59.5
Thigh SAT/area	0.47 ± 0.12	0.45 ± 0.13
Total cholesterol (mmol/L)	4.5 ± 1.0	4.7 ± 1.3
Triglycerides (mmol/L)	1.6 (1.1)	1.4 (1.1)
Waist circumference (cm)	114.3 ± 10.9	106.5 ± 17.4*
Hip circumference (cm)	114.8 ± 9.6	104.9 ± 8.8**
Waist-hip ratio	1.00 ± 0.09	1.01 ± 0.09
Weight (kg)	106.8 ± 13.6	90.1 ± 23.4**
Years with diabetes	5 (9)	9 (7)*
Diabetes treatment n(%)		
OAD / NPH insulin ± OAD	11 (52%) / 10 (48%)	5 (28%) / 13 (72%)
Other medications		
Statins	10 (48%)	4 (22%)
Blood pressure lowering agents	8 (38%)	7 (39%)
Self-reported complications		
Macrovascular	2 (10%)	3 (17%)
Retinopathy	2 (10%)	3 (17%)
Nephropathy or microalbuminuria	2 (10%)	6 (33%)
Neuropathy	1 (5%)	3 (17%)
Diabetic foot ulcers	1 (5%)	0 (0%)
Others (ED, fatty liver, periodontitis)	5 (24%)	6 (33%)
Co-morbidities		
Astma/COPD	4 (19%)	4 (22%)
Psychiatric conditions	4 (19%)	1 (6%)
GI disease	5 (24%)	2 (11%)
Other endocrine disorders	2 (10%)	3 (17%)
Smoking	10	1
Parity in women		
0–2	11	4
3–5	0	5

For normally distributed parameters, means ± SD are given, and for comparisons we used Student’s *t-test*. For non-normally-distributed parameters, medians (interquartile ranges) are given, and for comparisons we used the Mann–Whitney *U* test. Median HbA_1c_-values are reported as NGSP-%/IFCC-mmol/mol with interquartile range in parentheses. *p < 0.05 and **p < 0.01. Parity was defined as the number of live-born children each woman had given birth to. *OAD *oral antidiabetic drugs metformin and/or sulfonylureas. *ED *erectile dysfunction, *COPD *chronic obstructive pulmonary disease, *GI *gastro-intestinal, *AU *arbitrary units, *SAT *subcutaneous adipose tissue

### Ethnic differences in adipose tissue insulin resistance and plasma fetuin-A concentrations

**Fig. 1:**
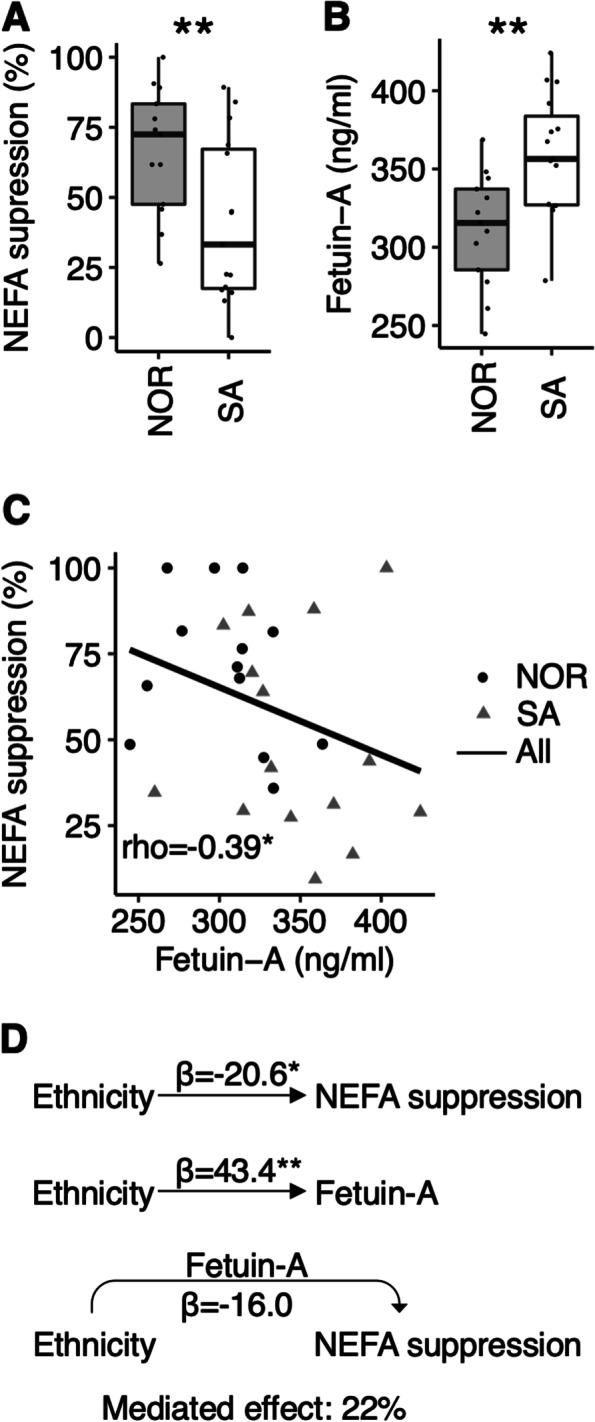
Ethnic differences in plasma fetuin-A levels and adipose tissue insulin resistance. **A** Insulin-induced suppression of lipolysis during the 2-step euglycemic hyperinsulinemic clamp, and **B** plasma fetuin-A concentrations in Norwegian (NOR) and South-Asian (SA) persons. **C** Correlations between plasma fetuin-A concentration and insulin-induced suppression of lipolysis during the 2-step euglycemic hyperinsulinemic clamp. The thick lines represents the correlation across all persons, and the stapled lines represent within-group correlations. **D** Mediation analyses on effects of plasma fetuin-A concentration on adipose tissue insulin resistance. *p < 0.05, **p < 0.01. AT-IR; Adipose tissue insulin resistance. NEFA; non-esterified fatty

NEFA-suppression during the clamp was lower in Pakistani than Norwegians (-41.2%, *p* = 0.045) (Fig. 1A). Plasma fetuin-A concentration was higher in Pakistani then Norwegians (9.2%, *p* = 0.008) (Fig. 1B). These differences were present both with and without adjustment for BMI. Plasma fetuin-A concentrations correlated negatively to %NEFA-suppression (Fig. 1C) during clamp (rho=-0.39, *p* = 0.039) across all participants. Within-group correlations were in the same directions but did not reach statistical significance for plasma fetuin-A concentrations vs. %NEFA-suppression (Norwegians: rho=-0.27, *p* = 0.371 and Pakistani: rho=-0.19 *p* = 0.491). Plasma fetuin-A concentration mediated 22% of the ethnic difference in NEFA-suppression during the clamp (Fig. 1D). Similar results were obtained with or without BMI correction (data not shown). In comparison, we observed no correlations between plasma fetuin-A concentrations and markers of skeletal muscle insulin resistance during clamping (GIR_40_ and GIR_400,_ data not shown).

### Sensitivity analyses

Because the ethnic groups differed in terms of T2DM-duration, HbA1c levels and T2DM treatments (Table 1), we constructed multiple regression models adjusting for these factors (Table 2). T2DM duration tended to correlate with NEFA-suppression (*p* = 0.091) whereas HbA1c levels or treatment regime showed no associations with NEFA-suppression (Table 2). The relationship between ethnicity and NEFA-suppression was not moderated by any of these factors (Table 2). T2DM duration, HbA1c levels or treatment regime were not associated with plasma fetuin-A levels and did not moderate the relationship between ethnicity and plasma fetuin-A levels (Table 2).

**Table 2:** Multiple regression models for sensitivity analyses

Model	β[95%CI]	*P*-value
*NEFA-suppression*		
**Model 1**		
(Intercept)	60.3[41.4,79.3]	< 0.001
Ethnicity	-27.3[-48.4,-6.3]	0.013
T2DM duration	1.7[-0.3,3.8]	0.091
**Model 2**:		
(Intercept)	56.2[-0.6,112.9]	0.052
Ethnicity	-23.2[-46.0,-0.5]	0.046
HbA1c	2.0[-5.3,9.2]	0.582
**Model 3**:		
(Intercept)	60.1[19.1,101.0]	0.006
Ethnicity	-20.2[-43.7,3.2]	0.088
OAD	12.3[-31.5,56.1]	0.568
OAD + NPH	11.2[-30.6,52.9]	0.586
*Fetuin-A*		
**Model 1**		
(Intercept)	307.9[277.6,338.1]	< 0.001
Ethnicity	45.9[12.3,79.5]	0.009
T2DM duration	-0.7[-3.9,2.6]	0.679
**Model 2**:		
(Intercept)	269.3[184.3,354.3]	< 0.001
Ethnicity	37.2[3.0,71.3]	0.034
HbA1c	4.6[-6.3,15.4]	0.393
**Model 3**:		
(Intercept)	308.7[246.5,371.0]	0.000
Ethnicity	42.5[6.8,78.2]	0.022
OAD	-6.2[-72.9,60.5]	0.850
OAD + NPH	-4.1[-67.5,59.4]	0.896

*CI *confidence interval, *OAD *oral antidiabetic drugs metformin and/or sulfonylureas, *NEFA *Non-esterified fatty acid,  *NPH *Neutral protamine Hagedorn insulin, *T2DM *type 2 diabetes mellitus

### Differences due to sex

We observed no sex differences in NEFA-suppression or plasma fetuin-A concentrations (data not shown).

### Ethnic differences in markers of adiposity and adipose tissue inflammation

**Fig. 2:**
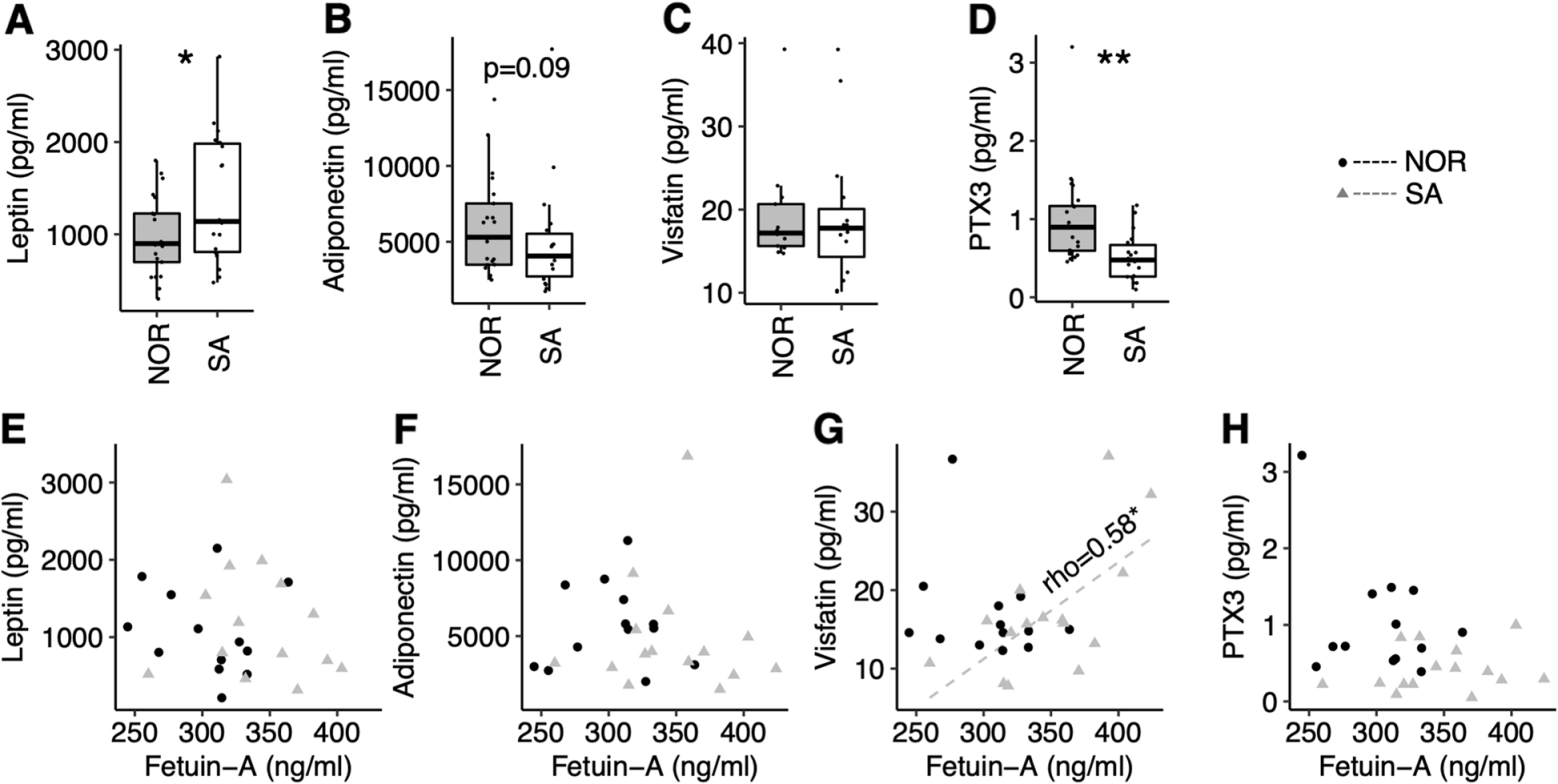
Ethnic differences in markers of adipose tissue inflammation.** A-D** Group comparisons. **E-F** Correlation scatter plots. *p < 0.05 and **p < 0.01. NOR; Norwegian. SA; South-Asian (Pakistani)

After adjustment for BMI, Pakistani patients displayed 21.0% higher plasma leptin (*p* = 0.029) (Fig. 2A) and 46.8% lower PTX3 (p = 0.0006) (Fig. 2D) levels compared to Norwegian. We observed no group difference in plasma visfatin levels (Fig. 2C), and a non-significant tendency to lower adiponectin levels (23.4%, *p* = 0.088) in Pakistani than Norwegian patients (Fig. 2B).

We observed no ethnic differences in the Spearman correlation between plasma fetuin-A and markers of adipose tissue inflammation (Fig. 2E-H), except for plasma visfatin levels, which correlated to plasma fetuin-A in Pakistani (rho = 0.58, *p* = 0.028), but not in Norwegian patients (Fig. 2G).

### Ethnic differences in markers of liver fat

**Fig. 3:**
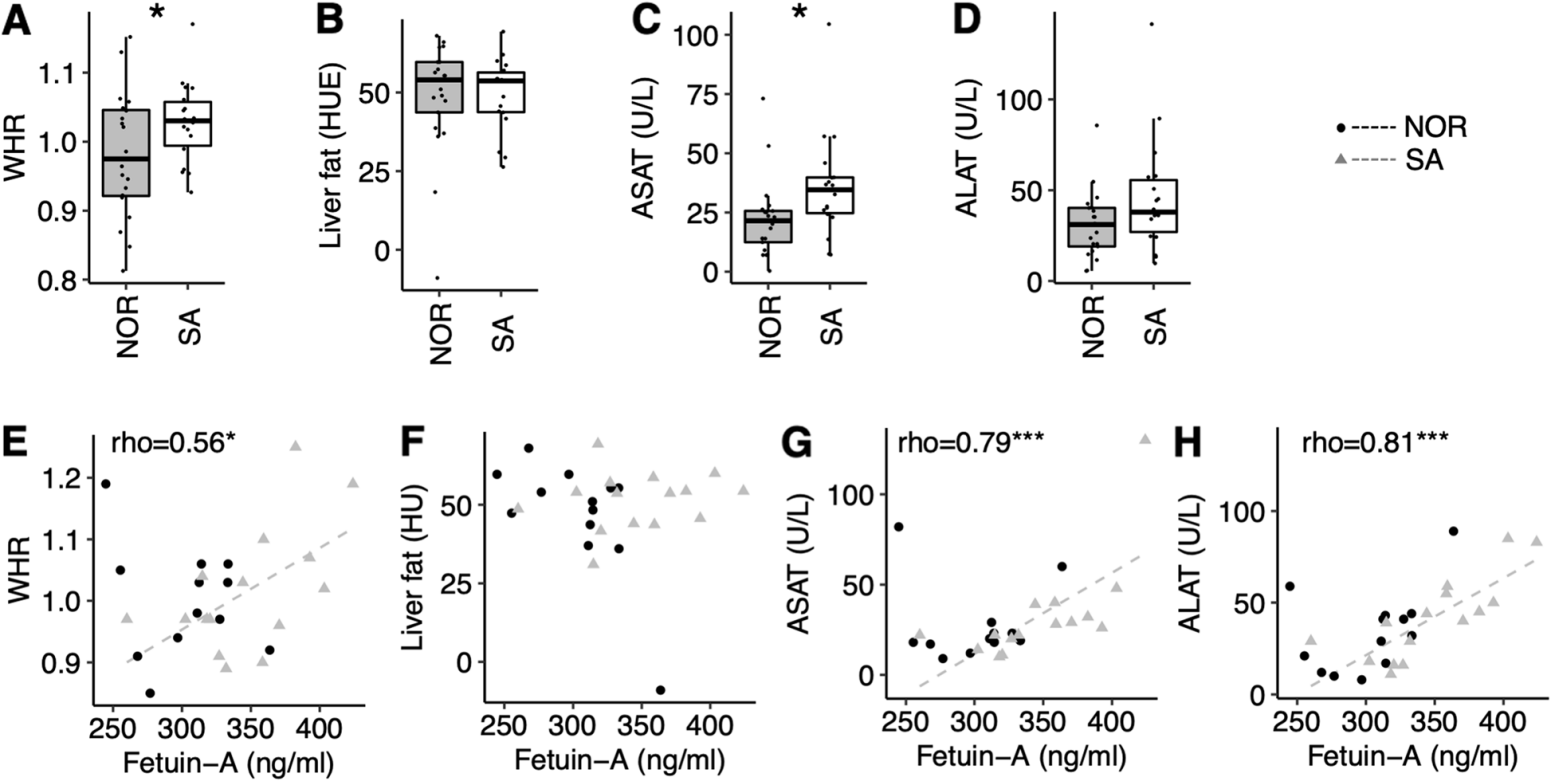
Ethnic differences in markers of liver fat content. **A** Group comparison of the waist-hip-ratio (WHR), **B** estimated liver fat content, and plasma levels of **C** ASAT and **D** ALAT. **E** Correlation between plasma fetuin-A levels and the waist-hip-ratio (WHR), **F** liver fat, **G** plasma ASAT and **H** ALAT levels. The grey stapled lines and the rho-values represent correlations in Pakistani patients. *p < 0.05, and ***p < 0.001. HUE = Hounsfield units. NOR; Norwegian. SA; South-Asian (Pakistani)

After adjusting for BMI, Pakistani exhibited higher waist-hip-ratio (5.4%, *p* = 0.025), similar liver fat content, and higher plasma aspartate aminotransferase (ASAT) (37.8%, *p* = 0.025) and alanine transaminase (ALAT) (18.0%, *p* = 0.090) levels than Norwegians (Fig. 3A-D). Plasma fetuin-A levels correlated positively to the waist-hip-ratio (rho = 0.56, *p* = 0.031), and the plasma ASAT (rho = 0.67, *p* < 0.001) and ALAT (rho = 0.74, *p* < 0.001) levels, in Pakistani patients only (Fig. 3E-H). We observed no significant correlations between plasma fetuin-A levels and liver fat content assessed by CT in any of the ethnic groups.

### Associations between plasma fetuin-A, and IL-1β, INF-γ, and IL-4 levels

**Fig. 4:**
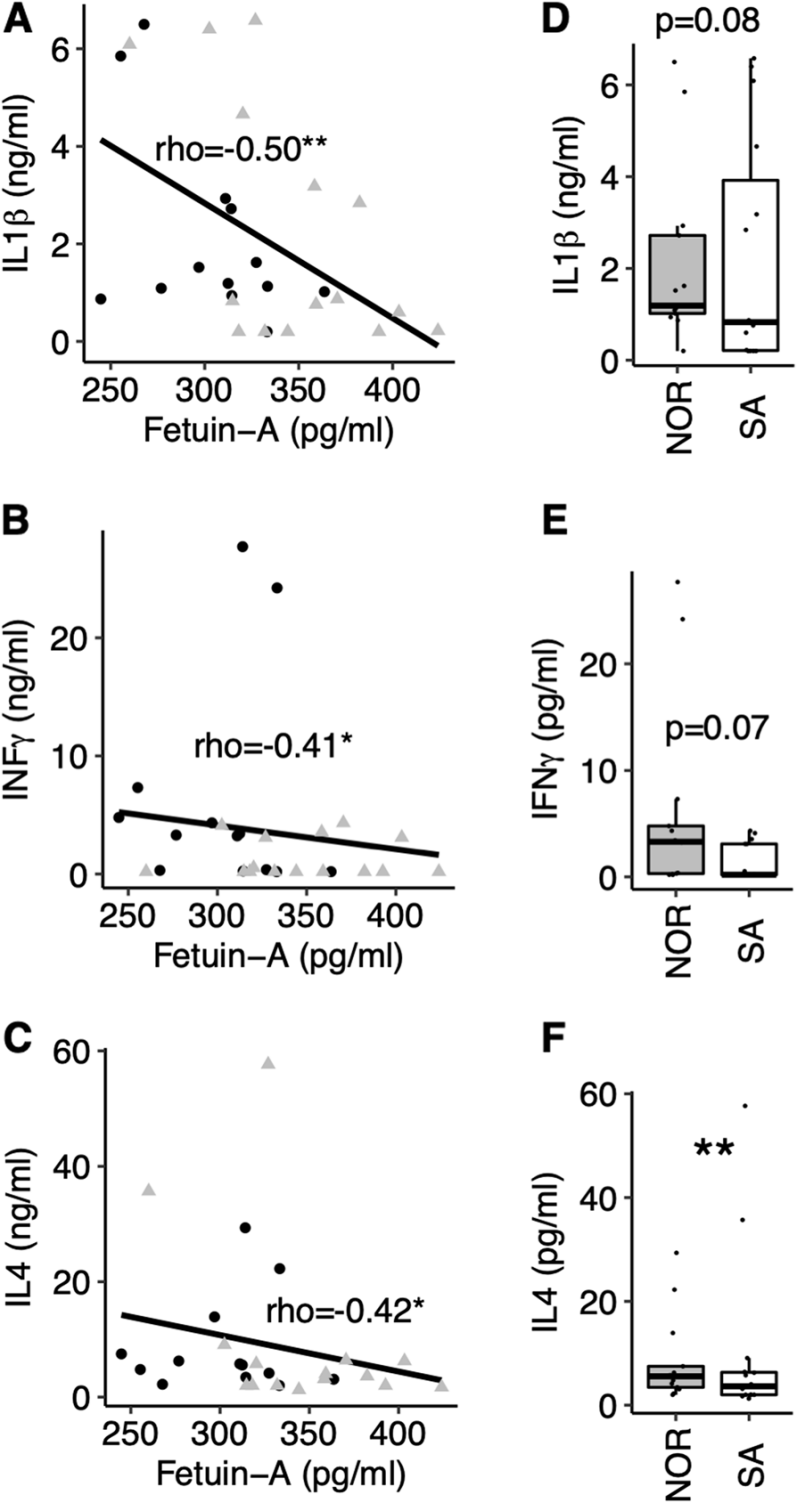
Plasma fetuin-A and IL-1β, INF-γ, and IL-4 levels. **A** Correlation scatter plots between plasma levels of fetuin-A and IL-1β, **B** INF-γ and **C** IL-4. **D** Group comparisons of plasma levels of IL-1β, **E** INF-γ and **F** IL-4. *p < 0.05. NOR; Norwegian. SA; South-Asian (Pakistani)

We observed negative correlations between the plasma concentrations of fetuin-A and IL-1β (Fig. 4A), INF-γ (Fig. 4B), and IL-4 (Fig. 4C), across the ethnic groups. Within-group correlations were in the same directions, but did not reach statistical significance for plasma fetuin-A concentrations vs. IL-1β (Norwegians: rho=-0.36, *p* = 0.231 and Pakistani: rho=-0.42 *p* = 0.114), plasma fetuin-A concentrations vs. INF-γ (Norwegian: rho=-0.27, *p* = 0.215 and Pakistani: rho=-0.02, *p* = 0.943), nor for plasma fetuin-A concentrations vs. IL-4 (Norwegian: rho = 0.20, *p* = 0.505 and Pakistani: rho = 0.31, *p* = 0.263).

After adjusting for BMI, Pakistani patients exhibited a non-significant tendency to lower plasma concentrations of IL-1β (-40.2%, *p* = 0.078) and INF-γ (-87.4%, *p* = 0.067), and lower IL-4 (-53.1%, *p* = 0.014) than Norwegian (Fig. 4D-F).

## Discussion

We observed that differences in adipose tissue insulin resistance between Pakistani and Norwegian patients with T2DM could be explained partly by differences in plasma fetuin-A concentrations. We also observed signs of more adipose tissue inflammation in Pakistani vs. Norwegian patients, and the inflammatory markers were associated with higher plasma levels of fetuin-A. The elevated plasma concentration of fetuin-A in Pakistani vs. Norwegian patients might relate to lower plasma levels of IL-1β, INF-γ, and IL-4 seen in the Pakistani patients.

Adipose tissue lipolysis is normally suppressed by insulin. Reduced suppression of lipolysis in response to insulin is thus an indicator of adipose tissue insulin resistance. Adipose tissue insulin resistance may contribute to lipid-induced insulin resistance in other tissues due to higher NEFA availability for the liver and can be a major pathogenic factor in T2DM [13, 16, 17]. However, an opposite relationship has been proposed where the liver may induce or worsen lipid-induced adipose tissue insulin resistance through increased secretion of fetuin-A that may bind saturated fatty acids and activate TLR4-signaling in adipose tissue, forming a vicious cycle to increase insulin resistance [13, 31].

We observed that young Pakistani patients with T2DM displayed reduced suppression of lipolysis in response to hyperinsulinemia during the clamp, suggesting that they have increased adipose tissue insulin resistance as compared to their Norwegian counterparts, in accordance with previous observations [9]. Furthermore, we show that these Pakistani patients with T2DM also display elevated plasma fetuin-A levels and that this difference in plasma fetuin-A levels might explain some of the observed difference in adipose tissue insulin resistance. In contrast, we observed no correlation between fetuin-A and measures of skeletal muscle insulin resistance. Thus, the effect of fetuin-A seems specific for insulin resistance in adipose tissue, as we have also indicated in a previous study [20].

Furthermore, despite differences in T2DM duration, HbA1c levels and T2DM treatment regimens between the two groups, adjusting for these factors in multiple regression analyses did not alter the associations between ethnicity, NEFA-suppression and plasma fetuin-A levels. We also note that body fat distribution is among the most important determinants of cardiometabolic risk and strongly impacts on insulin resistance of different organs [32]. However, we did not detect any difference in body fat percent of waist-hip ratio between the groups. Furthermore, low gluteofemoral fat mass strongly affect insulin resistance, independently of total fat mass [32], but we did not detect any group differences in thigh fat mass. We note that data obtained by body impedance analyses and CT-scans are not optimal to estimate body composition, and are greatly outperformed by magnetic resonance imaging (MRI) [33]. Thus, our data may give false negative results. We also note that differences in socioeconomic status may influence our results [34]. Unfortunately, we do not have data on the patients socioeconomic background.

We observed signs of more adipose tissue inflammation in Pakistani than Norwegian patients, as reflected in higher plasma concentrations of leptin [35], and lower plasma adiponectin and the anti-inflammatory PTX3 cytokine [36]. Moreover, increased plasma levels of the proinflammatory cytokine visfatin [37] were associated with higher plasma levels of fetuin-A in Pakistani patients, as also reported by others [38]. These observations are in line with in vitro studies showing that fetuin-A may increase proinflammatory cytokine production by activation of the TLR4 pathway in adipocytes [19]. Moreover, incubation with fetuin-A may inhibit the production of anti-inflammatory cytokines, such as adiponectin, and promote proinflammatory cytokines, such as IL-1β, TNF-α and IL-6 in human adipocytes and mice [24]. In vivo human studies have reported that enhanced levels of fetuin-A correlate with increased levels of several pro-inflammatory cytokines [19, 23]. Hence, we speculate that the increased adipose tissue insulin resistance in Pakistani than Norwegians might be explained by fetuin-A-induced inhibition of adipocyte insulin signaling by activation of inflammatory pathways [20, 21].

We also explored potential links to why plasma fetuin-A levels may be higher in Pakistani than Norwegian patients with T2DM. Pakistani patients had a higher waist-to-hip ratio than Norwegians, which correlated to increased plasma fetuin-A levels. However, we did not detect any ethnic differences in estimated liver fat content using CT-scans, nor any correlations between plasma fetuin-A levels and estimated liver fat content. These results are somewhat surprising because we expected a difference in liver fat content between the groups studied [39]. However, while CT-based diagnosis of hepatic steatosis is considered quite accurate, it is outperformed by dual gradient echo MRI (DGE-MRI) and 1 H-MRS in the diagnosis of non-alcoholic fatty liver disease (NAFLD) [33]. Our CT-derived estimation of liver fat content may explain why we did not observe a group difference, nor an association between liver fat content and fetuin-A levels in the present study. We observed higher plasma ASAT and ALAT concentrations in Pakistani patients, which correlated to increased plasma levels of fetuin-A.

Some researchers have suggested a cytokine-dependent down-regulation of fetuin-A expression in response to high plasma levels of IL-1β, INF-γ, and IL-4 [40]. Thus, we measured IL-1β, INF-γ, and IL-4 levels in plasma in our participants. We observed that plasma fetuin-A concentration was negatively correlated to plasma IL-1β, INF-γ, and IL-4 levels, consistent with previous reports [23, 40, 41]. We also observed lower plasma IL-4 levels, and tendencies to lower plasma IL-1β and INF-γ levels in Pakistani than Norwegian T2DM patients. Hence, we speculate that lower plasma levels of especially IL-4 may partly explain the difference in plasma fetuin-A levels between Pakistani and Norwegian T2DM patients. However, the exact mechanisms behind the links between fetuin-A and the pro/anti-inflammatory cytokines remain unknown.

The main weakness of this study is our limited sample size. Our results should be regarded as suggestive. Another weakness of this study is that we have no data on NEFA composition and dietary fat intake. Fetuin-A binds several types of NEFAs with different affinities, the strongest affinity is for saturated fatty acids like palmitic acid [15, 19]. Thus, differences in plasma NEFAs composition might influence the ability of fetuin-A to induce adipose tissue TLR4-signaling. Another weakness is that we do not have liver biopsies and we are unable to assess mRNA expression of fetuin-A directly. Furthermore, our study has a cross-sectional design and cannot imply causality. Future studies should be performed on a higher number of participants, monitor dietary intake, and perform more comprehensive lipidomics and cytokine profiling in blood and adipose tissue.

Taken together, we suggest a role for fetuin-A in the pathogenesis of T2DM by influencing adipose tissue inflammation and insulin resistance. Future studies should asses if elevated plasma fetuin-A levels may explain the high prevalence and early manifestation of T2DM in subjects with South Asian ethnicity living in Western countries.

## Data Availability

Data are available from the corresponding author on reasonable request.

## Abbreviations

ASAT: Aspartate aminotransferase
ALAT: alanine transaminase
anti-GAD: anti-glutamic acid decarboxylate
anti-IA2: anti-protein tyrosine phosphatase
BMI: body-mass index
ELISA: enzyme-linked immunosorbent assay
GIR40: glucose infusion rate at the low insulin infusion clamp step
GIR400: glucose infusion rate at the high insulin infusion clamp step
IL-1β: interleukin-1 beta
INF-γ: interferon-gamma
IL-4: interleukin-4
NEFA: non-esterified fatty acids
T2DM: type 2 diabetes mellitus
